# Ulcerative colitis in a transgender woman with a sigmoid neovagina: a case report

**DOI:** 10.1007/s00384-024-04676-x

**Published:** 2024-07-09

**Authors:** Anahita Sadeghi, Ehsan Bahrami Hezaveh, Ali Ali Asgari

**Affiliations:** Digestive Disease Research Institute (DDRI), Tehran University of Medical Sciences, Tehran, 14117-13135 Iran

**Keywords:** Ulcerative colitis, Colonoscopy, Sex reassignment surgery, Transsexualism, Case report

## Abstract

**Background:**

Sex reassignment surgery (SRS) is a necessary step in transitioning into the desired gender for male-to-female transgender individuals. This study focuses on a rare complication developed following SRS, aiming to highlight potential complications associated with this procedure.

**Case presentation:**

This report describes a 49-year-old transgender woman with a history of SRS who developed bloody diarrhea and neovaginal bleeding 10 years later. A colonoscopy revealed features compatible with ulcerative colitis, which was confirmed by a biopsy.

**Conclusions:**

The unpredictable clinical course of this phenomenon may prompt surgeons to reconsider the use of a rectosigmoid colon to create a neovagina. This case report underscores the necessity of long-term monitoring for gastrointestinal complications in transgender women post-SRS when a rectosigmoid colon segment is utilized for neovaginal construction.

## Introduction

Gender dysphoria is a condition defined by psychological distress that results from an incongruence between one’s sex assigned at birth and one’s gender identity [1]. For conversion to the preferred gender, sex reassignment surgery (SRS), which includes neovaginal construction, is a necessary step in the long course of transition for male-to-female transgender individuals [2]. There are a variety of surgical options for vaginal reconstruction, each with distinct positive and negative aspects. The use of the rectosigmoid for creating a neovagina has been proposed for more than a century, during which it has gained significant popularity as a method for vaginal reconstruction [3]. Rectosigmoid vaginoplasty is considered a safe and effective technique that provides satisfactory results in terms of both function and appearance [4, 5]. Ulcerative colitis (UC) is a chronic disease characterized by immune-mediated inflammation of the large intestine with the hallmark of bloody diarrhea [6]. UC development after vaginal reconstruction is a rare condition that has been reported previously in a few studies [7–13]. We report a case in which ulcerative colitis developed following neovaginal reconstruction surgery for male-to-female transition.

## Case report

A 49-year-old transgender woman with no surgical history underwent sex reassignment surgery, including sigmoid coloplasty and neovaginal construction at the age of 24. She developed bloody diarrhea and neovaginal bleeding 10 years later. She received antibiotics without improvement, and a colonoscopy showed ulcerative colitis (pancolitis), confirmed by biopsy, and characteristic features of UC such as crypt distortion, goblet cell depletion, and crypt abscesses were reported and immunohistochemistry (IHC) for cytomegalovirus (CMV) was negative in all samples. She was treated with mesalamine and azathioprine, and due to allergic reactions to azathioprine, she was switched to adalimumab and 6-mercaptopurine by her physician. She also had one course of hormone replacement therapy, but developed proximal deep vein thrombosis and was prescribed rivaroxaban. Due to a lack of testosterone following her previous gonadectomy, she undergoes regular evaluation using bone mineral density (BMD) measurements and is receiving calcium, vitamin D, and alendronate. She reported not being able to have any relationship with men after the surgery and never had sexual intercourse with her neovagina, but required several vaginal dilations for stricture formation. A transvaginal examination was not possible due to severe narrowing. The venereal disease research laboratory test was reported as negative.

**Fig. 1:**
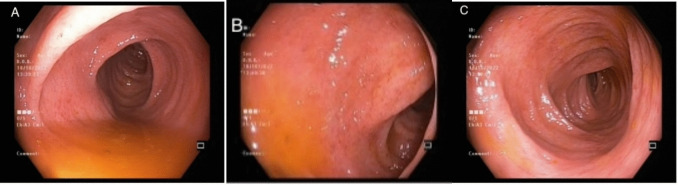
Endoscopic images from colonic mucosa showing loss of vascularity, contact bleeding, mucosal erythema, and multiple erosions. **A** and **B**: Descending colon, **C**: transverse colon

She presented to our center with loose, bloody stool six times per day. We performed a colonoscopy that revealed active ulcerative colitis in all colon segments, indicating a pancolitis with a Mayo score of 2 (Fig. 1). Pathological examination showed chronic active colitis with crypt distortion and cryptitis that confirmed the diagnosis. Adalimumab antibody level was 230 g/L, and the drug level was normal, indicating a secondary loss of response to anti-tumor necrosis factor (TNF). She was switched to tofacitinib. Additionally, she was referred to a surgeon for neovaginal removal to address the ongoing intermittent neovaginal bleeding, persistent inflammation, and stricture that could increase the risk of neovaginal cancer. Nevertheless, she has not agreed to the surgery. However, because the patient has refused revision surgery, we consider alternative surveillance methods and plan to use imaging techniques, such as magnetic resonance imaging, to obtain detailed information about the structure and any potential changes without the need for invasive procedures in follow-up.

**Fig. 2:**
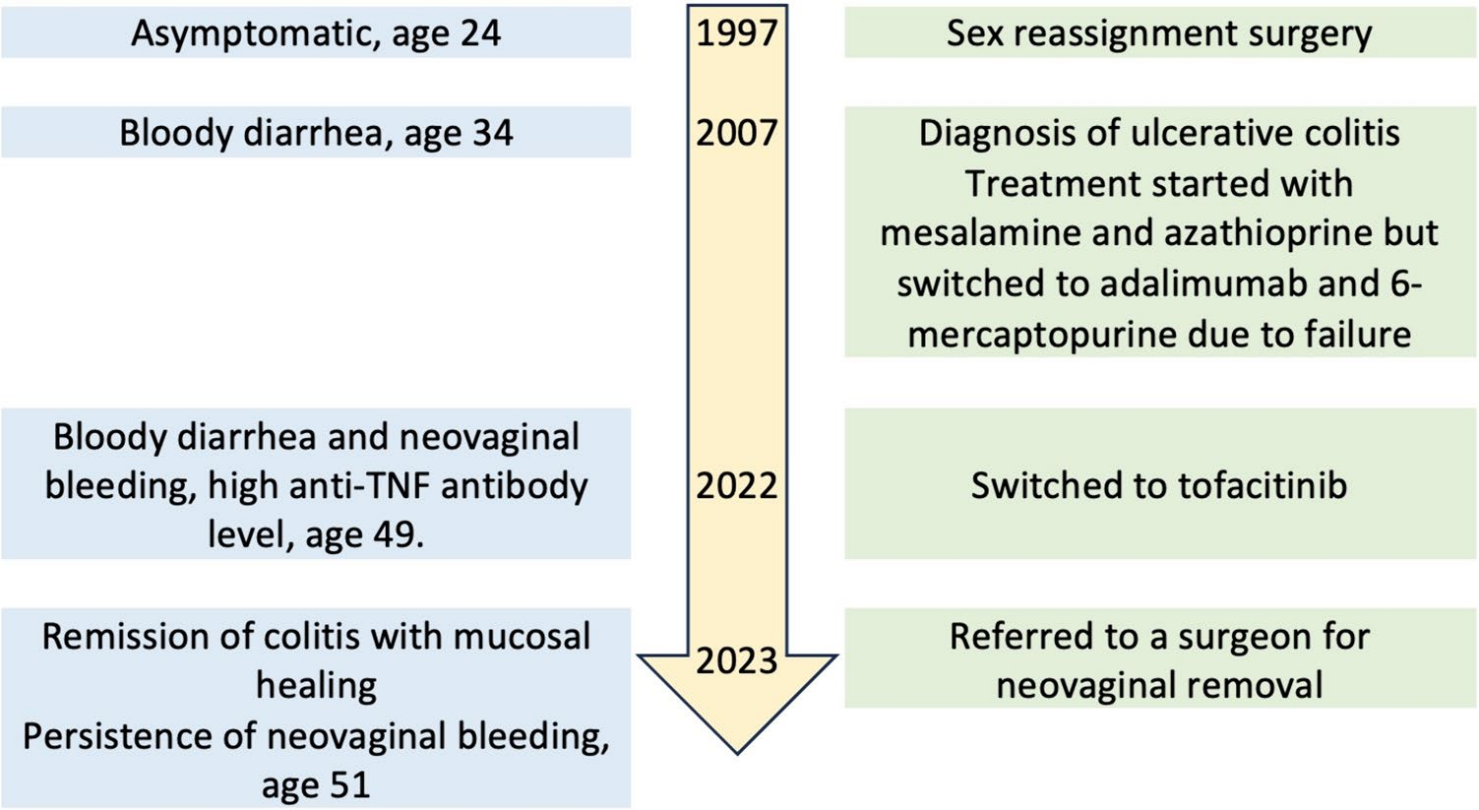
Timeline of historical and current information: sex reassignment surgery to mucosal healing. At the age of 24, the patient underwent sex reassignment surgery. At 34, the patient developed bloody diarrhea and was diagnosed with ulcerative colitis. Treatment with 5-ASA and azathioprine was switched to two different biologics due to IBD flares. Throughout this period, there were several flares. When the patient was 49, they were referred to our care due to an unresponsive IBD flare and were prescribed tofacitinib. Neovaginal removal was recommended due to a severe stricture that could not be evaluated. Currently, mucosal healing of the colon has been achieved; however, re-surgery is highly recommended due to neovaginal bleeding. (IBD inflammatory bowel disease, UC ulcerative colitis)

She has been symptom-free for 2 years and successfully tolerated the anti-TNF treatment without any adverse effects. A follow-up colonoscopy and pathology examination revealed mucosal healing; however, she still has occasional bleeding from the neovagina. The plan is to continue the current treatment, monitor her response and adverse effects, and also consult her for neovaginal removal. The timeline of historical and current information of the patient was shown in Fig. 2.

## Discussion

Reconstruction of neovagina has been used for treating various conditions, including congenital vaginal agenesis, transsexualism, cloacal anomalies, pelvic neoplasms, and certain injuries to the genitalia. Rectosigmoid reconstruction of the neovagina results in appropriate measures for sexual intercourse, physiological lubrication, and a low rate of adverse events [3, 14, 15]. Although using rectosigmoid for neovaginal construction is a widely used technique in SRS, the Penile Flap Inversion Vaginoplasty (PIV) is a safe gender-affirming surgery with minor complications [16].

Concurrent manifestations of ulcerative colitis in the colonic neovagina and remaining colon have been reported previously. In a 1992 study, a 26-year-old transgender individual was discussed with bloody vaginal discharge and diarrhea 10 years after sigmoid vaginoplasty for sex reassignment [9]. In another case presented in 2013, a teenage female with the XY genotype and a history of vaginoplasty at 4 months of age because of gonadal dysgenesis simultaneously presented similar vaginal and colonic symptoms [13]. We were not able to examine the vagina due to severe stenosis; however, the concurrent clinical presentation of bloody diarrhea and neovaginal bleeding confirms the hypothesis that a systemic predisposition to circulatory antigens triggers inflammation in both the colon and colonic neovagina [7].

After azathioprine failed due to allergies, our patient’s primary physician switched her to 6-mercaptopurine. However, she still had symptoms and needed adalimumab and methotrexate (MTX) as second-line therapy. When she came to our center, she was not in remission. We started tofacitinib because she had antibodies against the biological agent. This agent improved her symptoms. The response to treatment varies in the few case reports of similar presentations. In another report, a 60-year-old woman with a history of vaginal reconstruction due to congenital vaginal agenesis experienced bloody diarrhea and vaginal bleeding. The patient’s symptoms rapidly improved following the use of topical mesalamine [11]. Another study described a child who developed UC following vaginoplasty for the persistence of the cloaca. The authors stated that despite the use of various medications, such as azathioprine, infliximab, and methotrexate, the patient ultimately underwent subtotal colectomy due to the diagnosis of toxic megacolon [8]. In a letter to the editor discussing a patient with a history of vaginoplasty because of vaginal agenesis, coincident UC development in the sigmoid neovagina and the remaining colon occurred. They reported failure to respond to multiple drugs, including 5-amino salicylic acid, azathioprine, tacrolimus, and vedolizumab [12].

Ulcerative colitis can develop in both the colon and neovagina following vaginoplasty, regardless of the reason for the surgery. The clinical course of this phenomenon is highly unpredictable and varies from simple topical treatment to colectomy and resection of the neovagina. This prompts the question of whether a colonoscopy is necessary before the use of the rectosigmoid colon to create a neovagina. There is no recommendation regarding colonoscopy before creating a neovagina for individuals under the age of 45. A detailed history and physical examination of the patient, and individualized clinical decision-making, are mandated. Little is known about the incidence of neovaginal malignancies, but surveillance of a sigmoid neovagina is essential for early detection and timely intervention [17].

This study provides a detailed account of a rare condition that can arise after sex reassignment surgery, including its clinical course and endoscopic findings. However, there are some limitations that underscore the need for cautious interpretation of the findings. Firstly, the nature of a case report inherently limits the generalizability of the findings, necessitating individualized clinical judgment. Secondly, access to patient data from the initial surgery and subsequent ulcerative colitis diagnosis was unattainable. Lastly, the patient’s decision against neovaginal removal surgery precluded the possibility of evaluating the efficacy of potential treatments for vaginal bleeding.

In this case, we discussed a transgender woman who showed symptoms of ulcerative colitis (UC) 10 years after undergoing sex reassignment surgery (SRS). The emergence of ulcerative colitis a decade after SRS in this patient highlights an unexpected risk, indicating that the rectosigmoid technique may make individuals more susceptible to delayed complications. Clinicians should explore alternative approaches for creating a neovagina that could potentially reduce such risks. Additionally, conducting thorough preoperative assessments, including comprehensive gastrointestinal evaluations, could be considered to detect any pre-existing conditions that could impact surgical outcomes. This case report emphasizes the importance of taking a cautious and personalized approach to patient care and maintaining vigilance during the long-term postoperative period.

## Data Availability

The data supporting the conclusions of this study are not publicly available due to reasons of sensitivity and are available from the corresponding author upon reasonable request.

## Declarations

### Patient perspective

After a thorough evaluation and discussion with the patient regarding the potential risks and benefits, the patient expressed an inability to undergo further surgery. The treatment received was considered satisfactory by the patient, indicating a positive outcome in addressing the initial clinical symptoms. The decision was made to prioritize the patient's immediate health concerns over reconstructive surgery. The patient has been informed that the option for reconstructive surgery remains open for future consideration, once the present health challenges have been addressed, and close follow-up is recommended.
